# 

**Published:** 1817-02

**Authors:** 


### CATALOGUE OF MEDICAL BOOKS.

An Improved Method of Treating Strictures in the Urethra; by Thomas Whately, Member of the Royal College of Surgeons in London. Third Edition, with Additions. 8vo.

Transactions of the Medical Society of London. Vol. I. Part II. 8vo.

The Institutions of Physiology; by J. F. Blumenbach. Translated from the last Edition, and supplied with numerous Notes, by John Elliotson, M.D. 8vo.

Suggestions for the Improvement and Mitigation of Epidemic and Pestilential Diseases, comprehending the Abolition of Quarantines and Lazarettos; with some opportune Remarks upon the Danger of Pestilence from Scarcity, &c. &c. By Chas. Maclean, M.D. &c. 8vo.

Practical Observations in Surgery and Morbid Anatomy; illustrated by Cases, with Dissections and Engravings. By John Howship, Member of the Royal College of Surgeons, &c. &c. 8vo.

The Anatomy of the Human Ear; illustrated by a Series of Engravings of the Natural Size; with a Treatise on the Diseases of that Organ, the Causes of Deafness, and their Proper Treatment. By the late John Cunningham Saunders. 8vo. Coloured or plain.

The Annals of Medicine and Surgery; or, Records of the occurring Improvements and Discoveries in Medicine and Surgery, and the immediately connected Arts and Sciences. Vol. I. 8vo.

The Anatomy of the Horse; accompanied with a Series of Engravings, representing the Bones and Muscles of the Horse, in their systematical Arrangement; extracted from different Authors. By George Kirtland. No. 1. Coloured or plain.

A Catalogue of Books in Anatomy, Medicine, Surgery, Midwifery, Chemistry, Botany, &c. &c. New and Second-hand, for 1816-17. Sold by Anderson and Chase, 40, West Smithfield, London. 12mo.

Examinations of the Objections made in Britain against the Doctrines of Gall and Spurzheim; by J. G. Spurzheim, M.D. 8vo.

---

### *NOTICES TO CORRESPONDENTS.*

*Communications are received from various writers, which will be noticed and attended to.*

Dr. Balfour *must allow us to shorten his Answer, as a considerable part is not at all relevant to the controversy. If Dr. B. will permit us, he may rest assured, that the curtailment shall be made by the reviewer of his first paper.*

